# Rapid workflow of mMR PET list-mode data processing using CUDA

**DOI:** 10.1186/2197-7364-2-S1-A42

**Published:** 2015-05-18

**Authors:** Pawel Markiewicz, Kris Thielemans, David Atkinson, Simon Arridge, Brian Hutton, Sebastien Ourselin

**Affiliations:** University College London, Centre for Medical Image Computing, Translational Imaging Group, UK; University College London, Institute of Nuclear Medicine, UK

The purpose of this work is to provide a software solution which would enable very fast list-mode data processing offered by the massively parallel graphics processor units (GPU) while dealing efficiently with the common bottleneck of data transfers between hard disk storage, host (CPU) memory and device (GPU) memory. This software is dedicated to the PETLINK™ list-mode data format which is used by the Siemens’ hybrid Biograph Molecular MR (mMR) scanner. This software is developed as part of our platform for mMR 3D and 4D PET image reconstruction and will be freely accessible to the community. The aim is to perform real time processing (i.e. without perceivable delay) of the list-mode data during data transfers from hard disk to the CPU memory. The output of the processing includes: (1) The count-rate data (head curve: prompts, randoms and singles per second). (2) Plot of the variation of the centre of mass due to kinetics and motion (crude motion detection and quantification). (3) Projection movies (sagittal and coronal) for visual inspection for motion and data quality. (4) Crystal fan-sums for any time frame (used for randoms noise reduction). (5) Singles rate for each detector bucket (used for dead-time correction during normalisation). (6) Static and dynamic sinograms of span-1 and span-11. This workflow enables list-mode data processing synchronously with data transfer from disk to CPU memory. It opens a way for fast creation of multiple bootstrap realisations and multiple image reconstructions for a single dataset providing valuable insight into distributions of any statistic used in the development of robust image bio-markers. Also, the proposed workflow is advantageous for list-mode image reconstruction.

